# DDX41: exploring the roles of a versatile helicase

**DOI:** 10.1042/BST20230725

**Published:** 2024-02-13

**Authors:** Lacey Winstone, Yohan Jung, Yuliang Wu

**Affiliations:** Department of Biochemistry, Microbiology and Immunology, University of Saskatchewan, Saskatoon, SK S7N 5E5, Canada

**Keywords:** AML, DDX41, Helicase, innate immunity, MDS, ribosome biogenesis, R-loop, splicing, translation

## Abstract

DDX41 is a DEAD-box helicase and is conserved across species. Mutations in DDX41 have been associated with myeloid neoplasms, including myelodysplastic syndrome and acute myeloid leukemia. Though its pathogenesis is not completely known, DDX41 has been shown to have many cellular roles, including in pre-mRNA splicing, innate immune sensing, ribosome biogenesis, translational regulation, and R-loop metabolism. In this review, we will summarize the latest understandings regarding the various roles of DDX41, as well as highlight challenges associated with drug development to target DDX41. Overall, understanding the molecular and cellular mechanisms of DDX41 could help develop novel therapeutic options for DDX41 mutation-related hematologic malignancies.

## Introduction

Helicases are a large family of enzymes that utilize nucleoside triphosphate hydrolysis for energy to unwind DNA, RNA, and DNA–RNA hybrids; remove proteins from DNA or RNA; and remodel chromatins. There are several ways in which they can be categorized. Based on substrates, they are divided into DNA or RNA helicases. Based on their direction on DNA or RNA, they are characterized as 5′→3′ or 3′→5′ helicases. Based on sequence motifs, helicases are classified into six superfamilies (SF1–SF6) [1]; SF1 and SF2 members contain at least seven conserved motifs (I, Ia, II, III, IV, V, and VI) and exist as monomers, while SF3–SF6 assemble into hexamers. Helicases are involved in virtually all aspects of nucleic acid metabolism, including replication, repair, recombination, transcription, splicing, chromosome segregation, and telomere maintenance [2–5].

DEAD-box helicases are the largest family in SF2, named after the Asp-Glu-Ala-Asp (DEAD) sequence in the helicase motif II. First identified by Patrick Linder in 1989 [6], they have been found in virtually all prokaryotes and eukaryotes. DEAD-box helicases possess non-processive and local dissociation activity on double-stranded RNA substrates [7]. Many of them have drawn high interest from researchers due to their engagement in several processes of RNA metabolism. Various diseases, including neurodegeneration, inherited genetic disorders, and cancer, have been associated with DEAD-box helicases [8,9].

**Figure 1.**
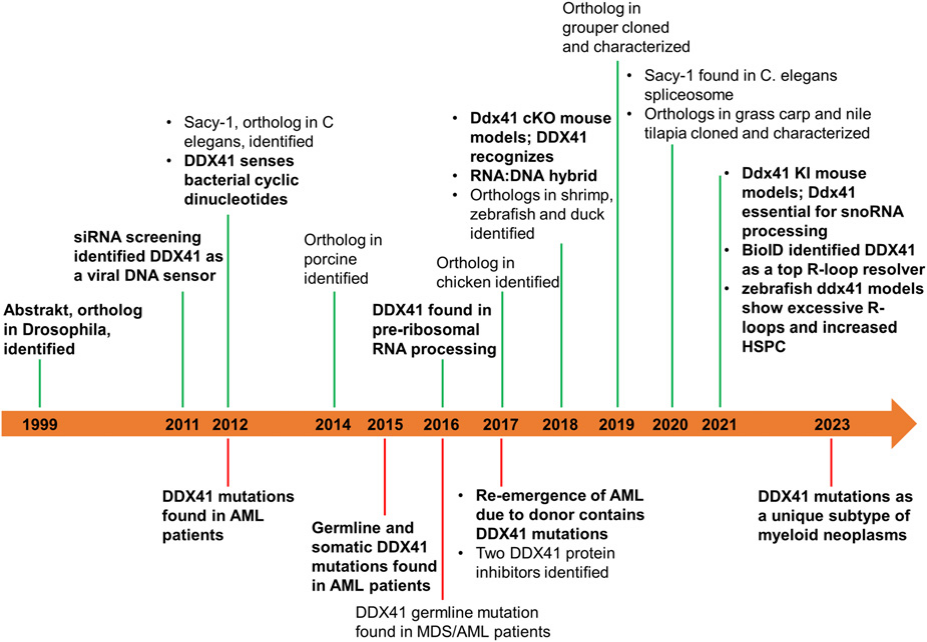
The discovery and characterization history of DDX41. The top represents basic sciences, and the bottom represents clinical or translational work. Significant events are bolded.

DDX41 is a DEAD-box helicase, which in humans is a 75-kDa protein with 622 amino acid residues. Abstrakt, DDX41's orthologs in *Drosophila* was discovered in 1999 [10]. Later, DDX41 was found in an extensive range of species, including *Caenorhabditis elegans* (Sacy-1) [11], porcine [12], duck [13], shrimp (*Penaeus monodon*) [14], chicken [15], grouper (*Epinephelus coioides*) [16], mandarin fish (*Siniperca chuatsi*) [17], grass carp (*Ctenopharyngodon idellus*) [18], Nile tilapia (*Oreochromis niloticus*) [19], zebrafish (*Danio rerio*) [20]*,* mouse and human [21] (Figure 1). Regardless of their minor difference in length among orthologs, DDX41 comprises a helicase core domain in the center, flanked by a nuclear localization signal domain in its N-terminus and a zinc-finger domain in its C-terminus.

## DDX41 associated diseases

Mutations in DDX41 have been associated with myeloid neoplasms (MNs), particularly myelodysplastic syndrome (MDS) and acute myeloid leukemia (AML). MDS is an incurable hematopoietic stem cell disorder characterized by ineffective hematopoiesis leading to bone marrow failure syndrome and progression to AML. In 2012, somatic DDX41 mutations were first reported in a study of sporadic AML syndrome [22] (Figure 1). In 2015, familial AML syndrome was characterized by long latency and germline mutations in the *DDX41* gene [23]. In 2016, five novel mutations, including missense and splicing mutations, were reported from families with MDS/AML [24–26]. The correlation between DDX41 mutations and MDS/AML was further confirmed in 2017 when two donor cell leukemia cases attributed to DDX41 mutations were reported [27,28]. In recent years, accumulating evidence has suggested that mutations in DDX41 cause MDS/AML [29–51], accounting for 2–5% of MN patients. Moreover, mutations in DDX41 are also associated with other hematologic malignancies, including acute lymphoblastic leukemia [52,53], chronic myeloid leukemia [38], acute erythroid leukemia [41], chronic myelomonocytic leukemia, non-Hodgkin lymphoma, Hodgkin disease and multiple myeloma [25].

**Figure 2.**
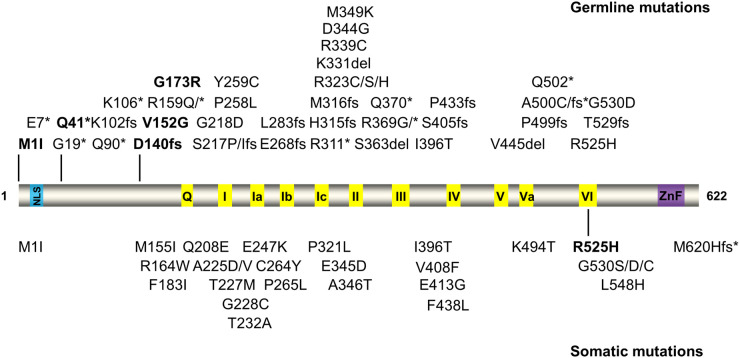
Summary of germline and somatic mutations reported in DDX41. The top are germline mutations, and the bottom are somatic mutations. The nuclear localization signal (NLS) domain is in blue, helicase motifs in yellow, and the zinc-finger (ZnF) domain in purple. fs, frame shift; *, nonsense truncation mutation. Only mutations with at least three patients (combined different cohorts) were shown. The hot spot mutants are in bold.

Current evidence suggests that DDX41 is a tumor suppressor gene. The predominant germline mutations are frameshift, truncation, and missense, such as D140fs (frameshift), Q41* (nonsense) , M1I, V152G and G173R [54] (Figure 2). Since most mutations occur in the N-terminus or helicase domain, they may lead to either truncation in protein or protein misfolding, subsequently causing a loss of function. Individuals born with germline DDX41 mutations are typically diagnosed in their sixties or later after somatic mutations occur. DDX41 somatic mutations are typically missense mutations. The most frequent somatic mutation is R525H; others include A225D, E247K, P321L, and G530S (Figure 2). The R525H mutation localizes in helicase motif VI, which is involved in ATP binding, and we found that it impacts ATP hydrolysis and helicase activity [55]. Notably, MDS/AML patients caused by DDX41 mutations show gender-specific prevalence. One study reviewing 277 MDS/AML patients affected by DDX41 mutations found that 222 patients (80.1%) were male, while 52 (18.8%) were female (the sex of 3 cases was not described) [56]. Overall, male-to-female patients are at a 4:1 ratio. Additionally, it is interesting that ethnicity affects the prevalence of different germline mutations. It is reported that M1I, D140fs (frameshift), G173R, and Q41* (nonsense) mutations occur exclusively in Western populations, whereas V152G, Y259C, A500fs, and E7* mutations are predominantly found in Asian populations [32,34]. Despite increasing clinical mutations reported in DDX41, its molecular pathogenesis, including sex and ethnic differences, remains unknown.

## Functions of DDX41

### Pre-mRNA splicing

Evidence from spliceosome and DDX41 itself supports that DDX41 is a spliceosome component. DDX41, known as Abstrakt at the time, was first found in the C complex that was purified and identified by two independent groups decades ago [57,58]. Later, DDX41 was found in the spliceosomal components (U1, U2, and U5) when V5-tagged DDX41 was overexpressed in HEK293 cells, followed by immunoprecipitation and mass spectrometry [23]. In 2020, Tsukamoto et al. [59] tagged endogenous SACY-1, the ortholog of DDX41 in *C. elegans*, with FLAG, coupled with immunoprecipitation and mass spectrometry, and found that it interacts with spliceosomal proteins, including proteins in the U1, U2, U5, B complex, C complex, and PRP19-CDC5L complex. Thus, DDX41 is considered a splicing protein.

**Figure 3.**
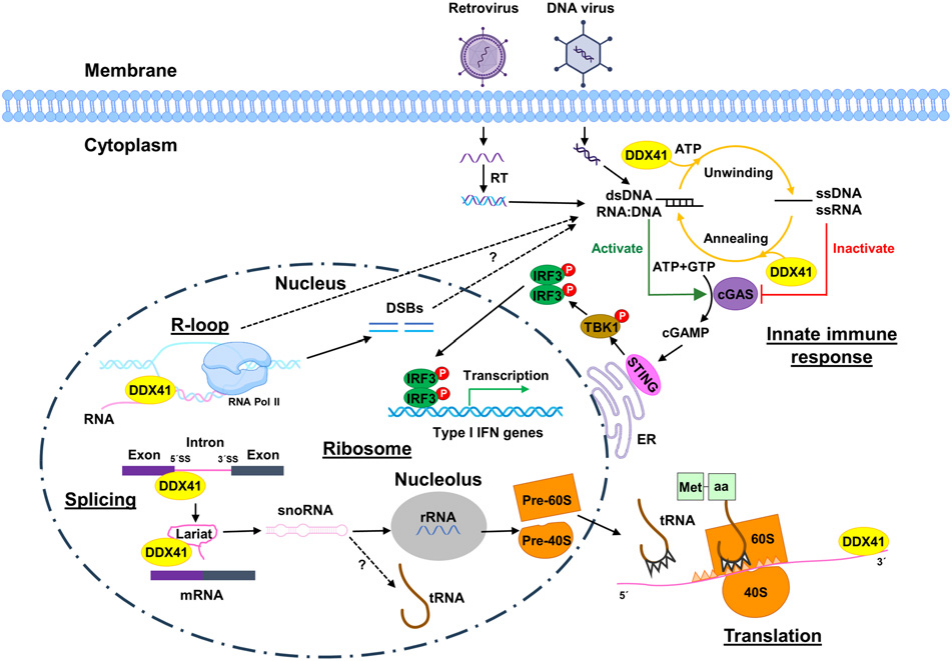
Potential functions of DDX41 protein. In the nucleus, DDX41 resolves R-loops during transcription, participates in pre-mRNA splicing, and snoRNA processing, which is essential for pre-60S and 40S assembly. In the cytoplasm, DDX41 senses DNA or RNA:DNA hybrid from invading DNA virus and retrovirus, respectively, and modulates nucleic acid forms (double and single-stranded) that activate and inactivate the cGAS-STING–TBK1–IRF3/7–IFN pathway. DDX41 also participates in translational regulation by binding on mRNA. Several vital pathways are not determined, such as whether R-loops and DSBs will leak from the nucleus to the cytoplasm to activate the cGAS–STING pathway and whether snoRNA will also regulate tRNA. A part of the figure was created with BioRender.

Despite DDX41 being found as a core mRNA splicing machinery component, its precise role has yet to be fully understood. The R525H mutant causes altered protein-protein interactions and increased exon skipping and retention [23]. Similarly, a depletion or mutations in SACY-1 in *C. elegans* result in global changes in transcriptome, including transcript abundance and splicing patterns [59]. Qin et al. [60] overexpressed DDX41 in HeLa cells, and RNA-seq revealed that DDX41 regulates transcription and alternative splicing of genes involved in tumorigenesis and immune response. Recently, Shinriki et al. [61] discovered that DDX41 mainly binds 5′ splice sites of coding RNA, indicating its role in forming activated spliceosomes (Figure 3). Nevertheless, further studies are warranted to address how DDX41 executes its role in pre-mRNA splicing, especially related to its helicase activity.

### Sensor in innate immunity

DDX41 senses invading nucleic acids, including DNA and RNA:DNA hybrids from viruses and bacterial cyclic dinucleotides, then triggers innate immune responses. In 2011, Yong-Jun Liu's group performed a siRNA screening for all 59 DExD/H-box helicases in myeloid dendritic cells and found that DDX41 is a DNA virus sensor [21]; this finding was supported by many orthologs of DDX41 found across species [12–20]. In 2012, Parvatiyar et al. [62] reported that DDX41 also acts as a bacterial cyclic di-GMP/AMP sensor that activates the type I interferons (IFNs) immune response. However, this result was observed in limited studies [20,63]. In 2018, Dr. Susan Ross and colleagues found that DDX41 recognizes RNA:DNA hybrids generated at the first step of reverse transcription from murine leukemia virus [64]. Upon recognizing viral nucleic acids, DDX41 may modulate the state of nucleic acids, namely double-strand and single-strand, which activate and inactivate cGAS, respectively, followed by STING-mediated signaling cascades to elicit an immune reaction (Figure 3). STING is highly expressed in immune-related cells, and the STING–TBK1–IRF3 pathway up-regulates the expression of type I IFNs and proinflammatory cytokines.

The cGAS–STING pathway is the central cellular cytosolic DNA sensor, allowing the innate immune response to infections, inflammation, and cancer. While cGas-complete-knockout (KO) mice display no overt developmental abnormality [65] and Sting-complete-KO mice are also viable [66], Ddx41-complete-KO mice are embryonically lethal [64,67]. These mouse models suggest that Ddx41 may be more crucial than cGas or Sting in mouse survival and cytokine immune response to a viral infection. Our recent work has demonstrated that DDX41 is essential for the cGAS–STING activation against DNA virus infections [55], suggesting that DDX41 functions upstream of cGAS and STING, which has recently been confirmed by another group [68]. We have also found that the annealing and unwinding activities of DDX41 are crucial to the on/off switch of cGAS [55]. DDX41 protein expression increases upon DNA virus infection, and then its annealing activity facilitates the formation of dsDNA, which prompts the cGAS–STING–type I IFN pathway. Once the virus is cleared, DDX41 expression is down-regulated, and then its unwinding function separates dsDNA into single-strand (ss)DNA, which inactivates the cGAS–STING–type I IFN pathway. Nevertheless, the regulation mechanisms of DDX41 dual activities and its cross-talk with other sensors, such as cGAS and IFI16, are largely unknown.

### Ribosome biogenesis

The creation of ribosomes is tightly controlled and is essential for protein production. DDX41 is involved in ribosome biogenesis. In 2016, Kadono et al. [69] showed that the DDX41 R525H mutation causes ribosomal stress and decreased protein synthesis. The R525H mutation is also associated with defective ribosome factors, indicating its role in pre-ribosomal RNA processing. In 2021, Dr. Daniel Starczynowski's lab generated Ddx41 conditional KO and knock-in mice, examined transcripts in leukemic stem/progenitor cells by RNA-seq, and found that small nucleolar RNAs (snoRNAs) are significantly overexpressed in Ddx41 KO animals [67]. Because snoRNAs guide small nuclear ribonucleoprotein complexes to catalyze chemical modifications of ribosomal RNA (rRNA) and transfer RNA (tRNA), dysregulated snoRNAs may have downstream impacts, including on rRNA modifications, pre-60S and pre-40S assembly, and protein production (Figure 3). Since most snoRNAs originate from lariats, DDX41 may facilitate the linearization of lariats by DBR1 (Debranching RNA Lariats 1) and degradation of lariats by nucleases, such as unwinding RNA stem-loops in the lariats, while the mutant R525H hinders this process. Whether increased snoRNAs influence tRNA's expression level and function remains unknown.

### Translational regulation

DDX41 is involved not only in ribosome biogenesis, but also in translation directly. Using siRNA to knockdown (KD) DDX41 in K562 cells, Dr. Hirotaka Matsui's group found that ribosome-associated genes are promoted after DDX41 KD, partly because these transcripts have significantly shorter transcript lengths and higher transcriptional and translational levels [70]. In addition, they found that transcripts with 5′-terminal oligopyrimidine motifs tend to be up-regulated when the DDX41 level is low. Previously, Peters et al. [71] reported that DDX41 negatively regulates the protein levels of p21, but not mRNA expression, through binding to the 3′UTR of the p21 mRNA. Collectively, DDX41 can regulate translation through two pathways: ribosome biogenesis throughout transcripts and as a trans-acting factor that binds to specific transcripts (Figure 3).

### R-loop metabolism

R-loops are three-stranded nucleic acid structures consisting of an RNA:DNA hybrid and a displaced ssDNA. DDX41 has been identified as an R-loop-associated protein using R-loop antibody S9.6 pulldown [72,73]. Zebrafish models suggest that DDX41 triggers an R-loop-mediated sterile inflammatory cascade [74]. DDX41 was identified as the top candidate for R-loop binding/resolving by an R-loop proximity proteomics approach [75]. Therefore, the correlation between DDX41 and R-loop is well established; however, whether dysregulated R-loop homeostasis is responsible for dysregulated innate response, genome instability, dysregulated splicing, ribosome biogenesis, and translation is yet to be determined.

### DDX41 senses R-loops in antiviral response

A decade ago, it was found that DDX41 KD in mouse and human cells inhibits the innate immune response and results in a defective activation of STING [21,62,76]; however, the underlying mechanism was unknown. Dr. Susan Ross’ group reported that DDX41 recognizes and binds RNA:DNA hybrids from retrovirus murine leukemia virus [64]. Using a zebrafish Ddx41 loss-of-function mutant, Dr. Teresa Bowman's group recently found that R-loop levels increase nearly two-fold in Ddx41 mutants [74]. Increased R-loops are also observed in DDX41-KD or by DDX41 inhibitor in K562 cells [61]. Recently, Dr. Karlene Cimprich's lab found that R-loops bind and activate cGAS in the cytoplasm [77], while our laboratory [55] and another group [68] found that DDX41 functions in the upstream of cGAS–STING. Collectively, it is reasonable to assume that DDX41 senses viral RNA:DNA hybrids and activates antiviral innate immune responses (Figure 3); however, it cannot be excluded that fragmented DNA from genomic instability could activate the cGAS–STING–IRF3–type I IFN pathway.

### DDX41 prevents R-loop-dependent genome instability

Accumulation of R-loops can lead to double-strand breaks (DSBs) and genomic instability [78]. Dr. Petra Beli's group used a BioID approach and identified DDX41 as a top R-loop resolver. Furthermore, they found that DDX41 proteins can unwind RNA:DNA hybrids *in vitro*, DDX41 localizes in the promoter regions, and DDX41 depletion leads to increased DSBs [75]. Increased R-loops and DSBs are also observed in DDX41 KD or by DDX41 inhibitors in K562 cells [61]. In addition, increased R-loops have been found in DDX41 KO human cardiomyocyte cell line AC16 cells [68]. Thus, evidence suggests DDX41 resolves R-loops and prevents R-loop-dependent genomic instability (Figure 3).

### DDX41 regulates R-loops in splicing, ribosome biogenesis, and translation?

R-loops have been implicated in pre-mRNA splicing [79], ribosome biogenesis [80], and translation [81]. In parallel, DDX41 is involved in pre-mRNA splicing [23,59,61], ribosome biogenesis [67,69,70], and translation [71]. However, the precise role of R-loops and DDX41 in these processes remains to be explored.

## Drug development targeting DDX41

Recent extensive cohort studies revealed that mutations in DDX41 account for 2–5% of patients with MDS/AML [82]. DDX41 germline mutations explain ∼80% of known germline predisposition to MNs in adults, and the life-long risk is ∼50% [44]. Venetoclax (Ven) in combination with hypomethylating agents (HMA), such as azacitidine or decitabine, have been widely adopted as a standard of care for patients with high-risk MDS and newly diagnosed AML. MD Anderson Cancer Center reported that 151 MDS/AML patients with DDX41 variants have ∼85% response rate for Ven/HMA therapy [31]. The median overall survival (OS) of patients with treatment-naïve AML or MDS is 49 and 71 months, respectively. In the 33% of patients receiving hematopoietic stem cell transplantation, the 2-year OS is ∼83% [31]. The University of Kansas Medical Center reported similar results when they studied 42 AML patients with DDX41 variants [83]. Mayo Clinic reported all 12 DDX41-mutated MDS/AML patients respond to Ven and HMA and achieve a complete response, including 80% deep response (no minimal residual disease) [45]. An Australian group reported that treating DDX41-mutated MDS and low blast count AML with azacitidine alone or with lenalidomide results in favorable outcomes [84]. However, given that both DDX41 alleles mutations account for the majority of MNs, germline alone and somatic mutation alone are also found in MNs [44], where the somatic missense mutation R525H is found in over 70% of patients. Mouse models also suggest not only biallelic mutations in DDX41, but also monoallelic DDX41-mutant affects hematopoiesis [67]. Therefore, additional options such as targeting mutant DDX41 (e.g. R525H) and synthetic lethality (SL) approach are sought to treat DDX41 mutant MDS/AML patients.

Using patients’ data in The Cancer Genome Atlas, we have previously examined the expression of DDX41 in 24 different cancer types and found that DDX41 is up-regulated in most cancers [85]. In addition, we found that the predominant mutant DDX41 R525H protein has reduced ATP hydrolysis and DNA unwinding activities but retains normal strand annealing activity that causes excessive innate immune activation [69]. These observations led to the developing specific inhibitors for DDX41 wildtype (WT) or R525H proteins. Yoneyama-Hirozane et al. [86] purified human DDX41 WT and the R525H mutant proteins, performed a high-throughput screening with ATPase assay, and identified two DDX41 inhibitors, named DDX41inh-1 and DDX41inh-2. These two inhibitors suppress cell growth of HeLa and K562 in a concentration-dependent manner, and DDX41inh-2 induces DNA replication stress, mitotic abnormalities, and DNA damage in post-mitotic cells [61]. However, their safety and therapeutic efficacy profiles in animal models and clinical trials have yet to be validated. Moreover, the somatic mutant R525H inhibits cell differentiation and cell growth of hematopoietic cells [61,67,69]. Thus, inhibitors targeting the DDX41 R525H protein but not the WT are wanted, likely through drug design based on their subtle structural differences.

Targeting DDX41 directly is quite challenging due to DDX41 being one of the 37 DEAD-box helicases in humans that share a similar structure [87]. Targeting the mutant DDX41 R525H is difficult because the mutant protein globally shares a similar structure with WT protein [55]. As such, SL becomes an alternative approach. Investigations of SL interactions can confer an effective strategy to exploit these hurdles and can be utilized for alternative potential cancer therapeutic options. Two genes are said to exhibit SL interaction if the loss-of-function of both these genes affects cellular viability, while neither has any effect on their own [88]. This concept facilitates the development of targeted therapies that selectively kill cancer cells while sparing normal cells. Using the SL concept, four poly (ADP-ribose) polymerase (PARP) inhibitors have been approved by the FDA for the treatment of BRCA1/2-mutant breast and ovarian cancers [89]. For DDX41-mutated MDS/AML, the SL approach possesses a strong rationale and potential because both alleles of DDX41 are mutated in most affected individuals. Instead of targeting DDX41 itself, we can target DDX41's SL partner, analogous to the case where PARP-1 inhibitors are used to treat BRCA1/2-mutated breast and ovarian cancer patients. However, no SL partner has been identified for DDX41 mutations. Using CRISPR screening and computational analysis of datasets from DepMap [90], we have discovered several genes exhibiting SL with DDX41. Currently, we are validating these genes.

## Conclusions

Current evidence suggests that DDX41 has multiple roles (Figure 3). Because it is physically localized in the spliceosome, concrete evidence supports that DDX41 participates in pre-mRNA splicing; however, its exact role remains to be defined. At least eight helicases have been implicated in pre-mRNA splicing, including DDX39, DDX46, DDX23, ASCC3L1, DHX16, DHX38, DHX8 and DHX15 [91]. Among them, DHX38 is in the C complex, ASCC3L1 and DDX23 are involved in promoting U6 base pairing and the disassembly of the U1 snRNA at the 5′ss; how DDX41 co-ordinates these helicases remains to be explored. In addition, it has been well-documented that DDX41 is implicated in R-loop metabolism. Because excessive R-loops cause DSBs and genomic instability, excessive R-loops, in turn, can activate the cGAS–STING–IRF3–type I IFN pathway that leads to chronic inflammation [77]. It is unclear whether excessive R-loops or fragmented DNA from DSBs will traffic from the nucleus to the cytoplasm and activate the cGAS–STING pathway. Lastly, DDX41 is also implicated in ribosome biogenesis, snoRNA process, and translation; however, its precise role is unclear. Nevertheless, it is not negligible that impaired splicing may lead to these RNA process and protein synthesis defects.

Although increasing cases of DDX41 mutations exist in clinical settings, how dysfunctional DDX41 results in hematolymphoid malignancies, such as MDS/AML, remains unknown. Though the overall prognosis of DDX41-related MDS/AML is favorable compared with most other types of *de novo* MDS/AML, and most patients respond to standard therapy [31,45,48,84,92,93]. However, the need for less toxic treatment is necessary due to the older populations with DDX41-mutated onset MN and high early mortality rate after bone marrow transplant. Hence, more investigations are needed to understand the structure and molecular mechanisms of DDX41. The use of artificial intelligence-aided drug discovery platforms and big data analytics for investigating correlations between aberrant DDX41 expression and DDX41-induced pathological conditions may facilitate the development of therapeutic options for patients with hematological malignancies caused by overexpression or dysfunction of DDX41.

## Perspectives

- Mutations in DDX41 have been associated with MNs, especially MDS and AML; however, its molecular pathogenesis remains unknown.
- DDX41 has many cellular roles, including pre-mRNA splicing, innate immune sensing, ribosome biogenesis, translational regulation, and R-loop metabolism.
- Despite significant progress has been made in understanding the functions of DDX41 since its discovery in 1999, several questions remain: (1) Since DDX41 protein is predominately localized in the nucleus, how does it traffic from the nucleus to the cytoplasm to sense a viral DNA? (2) Pre-mRNA splicing is a multi-step process; what is the exact role of DDX41 and its helicase activity? (3) How does DDX41 distinguish between invading viral and host DNA (and RNA:DNA hybrid) as a sensor? and (4) Are dysregulated R-loops responsible for all phenotypes, including genome instability, dysregulated innate immune response, splicing, ribosome biogenesis, and translation?

## Abbreviations

AML: acute myeloid leukemia
BioID: proximity-dependent biotin identification
DSB: double-strand break
HMA: hypomethylating agents
IFN: interferon
KD: knockdown
KO: knockout
MDS: myelodysplastic syndrome
MN: myeloid neoplasm
OS: overall survival
SL: synthetic lethality
snoRNA: small nucleolar RNA
tRNA: transfer RNA
WT: wildtype
